# Predictive performance of a competing risk cardiovascular prediction tool CRISK compared to QRISK3 in older people and those with comorbidity: population cohort study

**DOI:** 10.1186/s12916-022-02349-6

**Published:** 2022-05-04

**Authors:** Shona J. Livingstone, Bruce Guthrie, Peter T. Donnan, Alexander Thompson, Daniel R. Morales

**Affiliations:** 1 Division of Population Health and Genomics, University of Dundee, Mackenzie Building, Kirsty Semple Way, Dundee, UK; 2 Usher Institute, University of Edinburgh, Edinburgh, UK; 3 Division of Population Health, Health Services Research & Primary Care, University of Manchester, Manchester, UK; 4 Department of Public Health, University of Southern Denmark, Odense, Denmark

**Keywords:** Cardiovascular risk, Primary prevention, Risk prediction, QRISK3, Competing risk

## Abstract

**Background:**

Recommended cardiovascular disease (CVD) prediction tools do not account for competing mortality risk and over-predict incident CVD in older and multimorbid people. The aim of this study was to derive and validate a competing risk model (CRISK) to predict incident CVD and compare its performance to that of QRISK3 in UK primary care.

**Methods:**

We used UK linked primary care data from the Clinical Practice Research Datalink (CPRD) GOLD to identify people aged 25–84 years with no previous CVD or statin treatment split into derivation and validation cohorts. In the derivation cohort, we derived models using the same covariates as QRISK3 with Fine-Gray competing risk modelling alone (CRISK) and with Charlson Comorbidity score (CRISK-CCI) as an additional predictor of non-CVD death. In a separate validation cohort, we examined discrimination and calibration compared to QRISK3. Reclassification analysis examined the number of patients recommended for treatment and the estimated number needed to treat (NNT) to prevent a new CVD event.

**Results:**

The derivation and validation cohorts included 989,732 and 494,865 women and 946,784 and 473,392 men respectively. Overall discrimination of CRISK and CRISK-CCI were excellent and similar to QRISK3 (for women, C-statistic = 0.863/0.864/0.863 respectively; for men 0.833/0.819/0.832 respectively). CRISK and CRISK-CCI calibration overall and in younger people was excellent. CRISK over-predicted in older and multimorbid people although performed better than QRISK3, whilst CRISK-CCI performed the best. The proportion of people reclassified by CRISK-CCI varied by QRISK3 risk score category, with 0.7–9.7% of women and 2.8–25.2% of men reclassified as higher risk and 21.0–69.1% of women and 27.1–57.4% of men reclassified as lower risk. Overall, CRISK-CCI recommended fewer people for treatment and had a lower estimated NNT at 10% risk threshold. Patients reclassified as higher risk were younger, had lower SBP and higher BMI, and were more likely to smoke.

**Conclusions:**

CRISK and CRISK-CCI performed better than QRISK3. CRISK-CCI recommends fewer people for treatment and has a lower NNT to prevent a new CVD event compared to QRISK3. Competing risk models should be recommended for CVD primary prevention treatment recommendations.

**Supplementary Information:**

The online version contains supplementary material available at 10.1186/s12916-022-02349-6.

## Background

Cardiovascular disease (CVD) is the leading cause of death globally, estimated to cause 17.9 million deaths per year, and is the top-ranked cause of disability-adjusted life-years in people over the age of 50 years [1]. Risk prediction tools are recommended by guidelines to target statin initiation for the primary prevention of CVD for people above a specified threshold of predicted risk. Reflecting growing evidence of statin effectiveness for CVD primary prevention and falling medication costs, risk thresholds have also fallen over time. Historically, 10-year thresholds of 20% were recommended for statin initiation, but current risk thresholds for statin initiation vary internationally with a 10-year risk threshold of 7.5% being used in current US guidelines compared to 10% in England and Wales and 20% in Scotland [2–4]. The clinical benefit of risk-stratified guidelines therefore relies upon the accuracy of the underlying risk prediction tool.

Since age is the strongest predictor of CVD, current recommended thresholds effectively recommend that all older people are offered statin treatment, although the age at which this happens will vary with other risk factors. However, existing risk prediction models in older age and in people with comorbidity may not be accurate because such individuals are more likely to die from non-CVD conditions and may gain less benefit from statins whilst being exposed to some risk of harm and treatment disutility [5, 6]. Cox proportional hazard models for estimating the effects of variables on the hazard of the event occurrence are frequently used statistical methods in survival analysis. Survival analyses where data are censored typically assume that those lost to follow-up have the same risk of the outcome as those who remain in follow-up. This is clearly a false assumption in those who die and for those at high risk of dying from non-CVD causes. A competing risk is an event whose occurrence precludes the occurrence of the primary event of interest such as non-CVD death in this setting, which will be present in older people and those with multimorbidity [7, 8]. This leads to systematic overprediction of CVD risk using standard Cox regression models, and alternative methods are required that account for competing risk such as Fine-Gray models [9].

In England and Wales, the National Institute for Health and Care Effectiveness (NICE) currently recommend the QRISK3 tool to predict CVD risk [10]. In external validation of QRISK3, we showed that discrimination in the whole population was excellent (C-statistic 0.865 in women, 0.834 in men) but was poor in important subgroups (e.g. C-statistic 0.611 in women aged 75–84, 0.585 in men aged 75–84). In analysis accounting for competing risk, QRISK3 significantly over-predicted compared to competing risk adjusted observed mortality in older people and in those with high comorbidity [11].

The aim of this analysis was to first derive and internally validate a tool to predict incident CVD events that accounts for competing risk of non-CVD death and second to compare this model’s predictive performance in men and women to that of the UK recommended QRISK3.

## Methods

### Data source and population

We performed a cohort study in a large population of patients in the UK Clinical Practice Research Datalink (CPRD) Gold database [12, 13]. CPRD-GOLD contain primary care electronic health records from the UK that have been collected by general practitioners and are broadly representative of the UK population. CPRD-GOLD contains data on recorded health conditions, prescriptions, laboratory measurements taken in primary care, lifestyle, and measurement values. Data within CPRD-GOLD can be linked to UK data on hospitalisation and death. To be included, patients had to be permanently registered with a general practice contributing up-to-standard data in CPRD-GOLD for at least 1 year and with linkage to hospital episodes statistics (HES) discharge and Office for National Statistics (ONS) mortality data, be aged ≥ 25 years and < 85 years with no prior history of CVD (on GP records or linked hospital records), and have no history of prior statin treatment. Cohort entry was the latest of these dates on or after 1 January 2004. Cohort exit was the date of the earliest of first CVD event, non-CVD death, prescription of a statin, deregistration from the general practice, date of the last data collection from the practice, or the end of the study on 31 March 2016. The study was approved by the MHRA Independent Scientific Advisory Committee for database studies (ISAC 16/248).

### Outcomes

A first CVD event was defined as the earliest recording of any fatal or non-fatal coronary heart disease (CHD), ischaemic stroke, or transient ischaemic attack. Fatal CVD events were identified from ICD-10 codes recorded in ONS death registration. Non-fatal events were identified either in GP records (using Read codes, the standard coding system used in UK general practice) or HES discharge diagnoses (ICD-10 codes). Read and ICD-10 codes defining outcomes are those used in QRISK3 derivation and have previously been published [11].

### Prediction model

The following variables were included from the QRISK3 model: age, ethnicity, deprivation, systolic blood pressure, body mass index, total cholesterol to high density lipoprotein cholesterol ratio, smoking, family history of coronary heart disease in a first degree relative aged less than 60 years, type 1 diabetes, type 2 diabetes, treated hypertension, rheumatoid arthritis, atrial fibrillation, chronic kidney disease (stage 3, 4, or 5), systolic blood pressure variability (standard deviation of repeated measures), migraine, atypical antipsychotics, corticosteroids, systemic lupus erythematosus (SLE), severe mental illness, HIV/AIDs, and erectile dysfunction diagnosis or treatment in men. Our population was based on the published QRISK3-2017 prediction model with some exceptions, namely (1) we chose a later cohort entry date (1 January 2004 rather than 1 January 1998); (2) we handled cholesterol missingness differently (if no values were available at baseline, QRISK3 derivation allowed cholesterol values from *after* the index date to be used if they were before any event; we only included values recorded before the index date to avoid using future information in prediction); and (3) we evaluated the Townsend deprivation score as the median of the vigintile (equal 20th) of score that an individual lived in, as individual values were not available. We included all covariates that were included in the QRISK3 model. Read and ICD-10 codes defining predictors in QRISK3 are not publicly available. We therefore developed our own code sets, and these and methods of data handling have previously been published [11].

### Comorbidity

For each patient at baseline, we additionally calculated a modified Charlson Comorbidity Index (CCI) based on primary care Read codes (modified in that CVD could not contribute to the score as all participants are CVD-free at baseline) using a published code set for this purpose [14]. CCI (grouped into 0, 1, 2, and 3+) was included in the competing risk model as a predictor of non-CVD death to examine whether this improved model performance.

### Missing data

As with QRISK3 derivation, patients with missing Townsend deprivation score were excluded from the cohort, those with missing ethnicity were assumed to be white, and multiple imputation was used for missing body mass index (BMI), total cholesterol to HDL cholesterol ratio (TC:HDL), systolic blood pressure (SBP), SBP variability, and smoking status assuming data was missing at random [11]. Multiple imputation included all predictor variables and the outcome. Multiple Imputation by Chained Equations was used to generate five imputed datasets [15]. Analyses of these datasets were combined using Rubin’s rules to give summary point estimates with confidence limits that reflect the added uncertainty associated with imputing missing values [16].

### Statistical methods

The study size was determined by the data available in CPRD, which was considered sufficient, and no formal power calculation was done [17]. Patients were randomly allocated to a fixed derivation and test dataset in a 2:1 ratio with the split balanced in terms of age and final event status. The derivation dataset was used to derive CRISK, a new Fine-Gray model to predict the 10-year risk of experiencing a CVD event accounting for the competing risk of non-CVD death. Separate models were estimated for men and women. The Fine-Gray model calculates the subdistribution hazard ratio that is the instantaneous risk of failure from the CVD event in subjects who have not yet experienced a CVD event, whilst simultaneously accounting for the occurrence of non-CVD death. Since we wished to explicitly compare prediction in a model accounting for competing risk versus QRISK3, we included all the same main effects and age interactions as in QRISK3, but we also accounted for non-CVD death as a second (competing) outcome. We also re-estimated fractional polynomial terms for continuous variables, selecting terms based on those performing best (as measured by the C-statistic) in balanced 10-fold cross-validation and showing consistency of model fit (AIC) across folds of the derivation data set. We then derived a further model (CRISK-CCI) which additionally included the CCI score in the model (categorised as 0, 1, 2, ≥ 3) as a validated predictor of total mortality [14]. Note that these models allow the cumulative incidence function (CIF) or probability of a CVD event occurring over time to be directly predicted. However, the subdistribution hazard ratios (sHRs) in the Fine–Gray models describe the direction but not the magnitude of the effect of predictors on the CIF. Also, the use of fractional polynomials and the inclusion of interactions with age further complicate their interpretation.

The performance of CRISK and CRISK-CCI was compared to QRISK3 in the independent validation dataset by examining discrimination and calibration of all models. Discrimination is the ability of the risk score to differentiate between patients who experience the event of interest during the study and those who do not. We used Harrell’s C-statistic to describe discrimination. A C-statistic of 0.5 indicates discrimination that is no better than chance, whereas a C-statistic of 1 indicates perfect discrimination [18].

Calibration refers to how closely the predicted and observed probabilities agree at group level. This was assessed by plotting the observed versus predicted risk for CRISK, CRISK-CCI, and QRISK3. Observed risk was estimated using the Aalen-Johansen estimator which accounts for competing mortality risk [19]. Plots were generated separately by sex, for all patients and for pre-specified subgroups of age and CCI based on summary statistics pooled across the imputed dataset.

### Examining patient reclassification

CVD guideline recommendations for primary preventive treatment use thresholds of predicted risk to classify patients as having a high enough risk of CVD to be offered treatment. We examined changes in patients recommended for treatment by CRISK-CCI and QRISK3, focusing on patients reclassified to be either side of the 20% (UK recommended threshold till 2014), 10% (current NICE recommended threshold), and 7.5% (plausible future) thresholds of predicted CVD risk. We described the characteristics of reclassified patients including the observed risks of CVD at 10 years and the number needed to treat to prevent one new CVD event assuming all people recommended for treatment take a statin having a relative risk reduction of 25% for new CVD events. All models were fitted in R, version 4.0.0, and STATA, version 11.2.

## Results

A flow chart for cohort identification is shown in Additional file 1: Fig. S1. There were 989,732 women and 946,784 men aged 25–84 in the derivation cohort and 494,865 and 473,392 respectively in the validation cohort. The baseline characteristics of each study population were similarly distributed in the derivation and validation cohorts (Table 1). Missing data was present for ethnicity (women 20.9%: men 35.5%), smoking status (women 20.2%: men 31.2%), SBP (women 17%: men 34.5%) and BMI (women 27.6%: men 41.6%), and more frequently for total cholesterol to HDL cholesterol ratio (women 84.9%: men 85%) and SBP variability (women 46.9%: men 74%) (Additional file 1: Table S1). Follow-up status at 10 years by sex, age, and co-morbidity in the derivation cohort are shown in Additional file 2: Table S2. In the derivation cohort, there were 14,150 incident cases of CVD observed in women in 2,865,660 years of follow-up (4.9 [95%CI 4.89–4.99] per 1000 person-years), compared to 17,689 incident cases in men in 2,632,804 years of follow-up (6.7 [95%CI 6.66–6.78] per 1000 person-years). CVD incidence rose progressively with age (Additional file 1: Table S3). The final sex-specific Fine–Gray models for the main outcome of interest (CVD) are reported in Additional file 1: Table S4 and Additional file 1: Table S5.

**Table 1:** Baseline data in the derivation and validation cohort

Characteristic	Women		Men	
	Derivation cohort ***N*** = 989,732	Validation cohort ***N*** = 494,865	Derivation cohort ***N*** = 946,784	Validation cohort ***N*** = 473,392
Smoking status recorded	789,562 (79.8)	394,819 (79.8)	651,127 (68.8)	325,555 (68.8)
Body mass index (BMI) recorded	716,715 (72.4)	358,418 (72.4)	552,983 (58.4)	276,680 (58.4)
Total cholesterol to HDL cholesterol ratio recorded	149,188 (15.1)	74,602 (15.1)	142,013 (15.0)	70,741 (14.9)
Systolic blood pressure (SBP) recorded	821,768 (83.0)	410,765 (83.0)	620,788 (65.6)	310,112 (65.5)
2 or more SBP readings recorded before baseline	525,966 (53.1)	262,696 (53.1)	245,671 (25.9)	122,884 (26.0)
Complete data for BMI, total to HDL cholesterol ratio, SBP, and smoking status	95,886 (9.7)	47,996 (9.7)	79,081 (8.4)	39,496 (8.3)
Age (years)	46.0 (15.3)	46.0 (15.3)	44.8 (13.9)	44.8 (13.9)
Townsend score	− 0.6 (2.8)	− 0.6 (2.8)	− 0.5 (2.8)	− 0.5 (2.8)
BMI (kg/m^2^)	25.9 (5.7)	25.9 (5.7)	26.6 (4.7)	26.6 (4.7)
Total cholesterol to HDL cholesterol ratio	3.7 (1.1)	3.7 (1.1)	4.4 (1.3)	4.4 (1.3)
Systolic blood pressure (mmHg)	125.4 (18.0)	125.4 (18.0)	131.1 (16.2)	131.1 (16.2)
Systolic blood pressure variability	10.0 (5.7)	9.9 (5.7)	10.3 (6.2)	10.3 (6.2)
Ethnicity				
Ethnicity recorded	783,626 (79.2)	391,224 (79.1)	610,093 (64.4)	305,385 (64.5)
White or not recorded	908,817 (91.8)	454,329 (91.8)	891,002 (94.1)	445,219 (94.0)
Indian	15,045 1.5)	7443 (1.5)	10,182 (1.1)	5140 (1.1)
Pakistani	6411 (0.6)	3139 (0.6)	4452 (0.5)	2222 (0.5)
Bangladeshi	1717 (0.2)	877 (0.2)	1403 (0.1)	742 (0.2)
Other Asian	9027 0.9)	4670 (0.9)	6636 (0.7)	3337 (0.7)
Black Caribbean	6369 (0.6)	3136 (0.6)	4455 (0.5)	2232 (0.5)
Black African	12,420 (1.3)	6384 (1.3)	8535 (0.9)	4287 (0.9)
Chinese	4390 (0.4)	2349 (0.5)	2325 (0.2)	1178 (0.2)
Other	25,536 (2.6)	12,538 (2.5)	17,794 (1.9)	9035 (1.9)
Smoking status				
Non-smoker	471,885 (59.8)	235,889 (59.7)	318,933 (49.0)	159,738 (49.1)
Former smoker	144,631 (18.3)	72,773 (18.4)	144,517 (22.2)	72,366 (22.2)
Light smoker	56,913 (7.2)	28,364 (7.2)	50,254 (7.7)	25,006 (7.7)
Moderate smoker	74,332 (9.4)	37,358 (9.5)	74,919 (11.5)	37,492 (11.5)
Heavy smoker	41,801 (5.3)	20,435 (5.2)	62,504 (9.6)	30,953 (9.5)
Family history of coronary heart disease in 1st degree relative < 60 years	65,264 (6.6)	32,360 (6.5)	50,139 (5.3)	25,098 (5.3)
Type 1 diabetes	2512 (0.3)	1240 (0.3)	3227 (0.3)	1616 (0.3)
Type 2 diabetes	11,336 (1.1)	5686 (1.1)	14,147 (1.5)	6930 (1.5)
Treated hypertension	77,148 (7.8)	38,796 (7.8)	55,397 (5.9)	27,371 (5.8)
Rheumatoid arthritis	8510 (0.9)	4192 (0.8)	3111 (0.3)	1613 (0.3)
Atrial fibrillation	5412 (0.5)	2787 (0.6)	7058 (0.7)	3562 (0.8)
Chronic kidney disease (stage 3, 4, or 5)	4636 (0.5)	2282 (0.5)	3789 (0.4)	1870 (0.4)
Migraine	78,530 (7.9)	39,162 (7.9)	27,765 (2.9)	13,706 (2.9)
Corticosteroid use	13,887 (1.4)	6787 (1.4)	7833 (0.8)	3991 (0.8)
HIV/AIDS	206 (0.0)	83 (0.0)	264 (0.0)	181 (0.0)
Systemic lupus erythematosus	1149 (0.1)	576 (0.1)	108 (0.0)	57 (0.0)
Atypical antipsychotic use	5663 (0.6)	2806 (0.6)	5511 (0.6)	2825 (0.6)
Severe mental illness	73,909 (7.5)	36,890 (7.5)	38,202 (4.0)	19,062 (4.0)
Erectile dysfunction diagnosis or treatment	NA (NA)	NA (NA)	26,099 (2.8)	13,165 (2.8)
Charlson comorbidity index score				
0	791,395 (80.0)	396,570 (80.1)	781,665 (82.6)	391,400 (82.7)
1	153,590 (15.5)	76,061 (15.4)	134,497 (14.2)	66,703 (14.1)
2	34,250 (3.5)	17,045 (3.4)	23,133 (2.4)	11,532 (2.4)
3+	10,497 (1.1)	5189 (1.0)	7489 (0.8)	3757 (0.8)

Mean (SD) or number (%)

### Discrimination of CRISK-CCI, CRISK, and QRISK3

In the validation cohort, overall discrimination of CRISK and CRISK-CCI were excellent and similar to QRISK3 (for women, C-statistic = 0.863 for CRISK vs 0.864 for CRISK-CCI vs 0.863 for QRISK3: for men C-statistic 0.833 vs 0.819 vs 0.832 respectively) (Table 2). Similar to QRISK3, discrimination for CRISK and CRISK-CCI varied by age group and CCI categories, with discrimination being best in the youngest (25–44 years) and least multimorbid (CCI = 0) groups and worst in the oldest (75–84 years) and most multimorbid (CCI = 3+) groups. For example, in women aged 75–84, C-statistic = 0.614 for CRISK vs 0.616 for CRISK-CCI vs 0.613 for QRISK3, and for men aged 75–84, C-statistic = 0.594 vs 0.570 vs 0.590 respectively.

**Table 2:** Discrimination and model fit of CRISK-CCI, CRISK, and QRISK3 for men and women in the validation cohort

	Women—Harrell’s C-statistic (95%CI)			Men—Harrell’s C-statistic (95%CI)		
	CRISK-CCI	CRISK	QRISK3	CRISK-CCI	CRISK	QRISK3
All patients	0.864 (0.859,0.869)	0.863 (0.858,0.869)	0.863 (0.858,0.868)	0.819 (0.815,0.824)	0.833 (0.828,0.837)	0.832 (0.827,0.836)
Age group						
Age 25–44	0.763 (0.745,0.781)	0.761 (0.743,0.779)	0.765 (0.747,0.783)	0.733 (0.720,0.746)	0.744 (0.731,0.757)	0.740 (0.727,0.753)
Age 45–64	0.713 (0.703,0.722)	0.710 (0.701,0.720)	0.708 (0.698,0.717)	0.661 (0.654,0.668)	0.683 (0.676,0.690)	0.679 (0.672,0.686)
Age 65–74	0.647 (0.637,0.658)	0.645 (0.634,0.655)	0.641 (0.631,0.652)	0.591 (0.581,0.600)	0.610 (0.600,0.619)	0.606 (0.596,0.615)
Age 75–84	0.616 (0.607,0.624)	0.614 (0.605,0.622)	0.613 (0.604,0.622)	0.570 (0.559,0.580)	0.594 (0.583,0.604)	0.590 (0.580,0.601)
CCI						
CCI 0	0.862 (0.855,0.868)	0.862 (0.855,0.868)	0.861 (0.855,0.868)	0.812 (0.806,0.817)	0.825 (0.820,0.831)	0.824 (0.818,0.829)
CCI 1	0.843 (0.833,0.854)	0.843 (0.833,0.854)	0.843 (0.833,0.854)	0.815 (0.805,0.826)	0.830 (0.820,0.841)	0.830 (0.819,0.840)
CCI2	0.787 (0.770,0.805)	0.788 (0.771,0.806)	0.789 (0.771,0.806)	0.704 (0.685,0.722)	0.729 (0.710,0.747)	0.728 (0.709,0.747)
CCI 3+	0.753 (0.725,0.781)	0.754 (0.726,0.782)	0.754 (0.726,0.782)	0.666 (0.636,0.695)	0.698 (0.668,0.727)	0.695 (0.665,0.724)

*CCI* Modified Charlson Comorbidity Index

### Calibration of CRISK-CCI, CRISK, and QRISK3

**Fig. 1:**
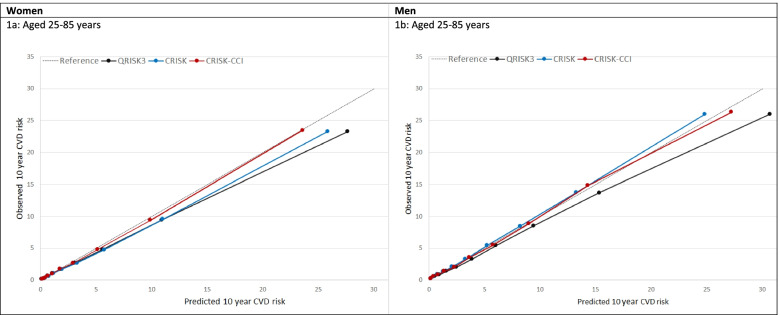
Calibration of the competing risk model with the Charlson comorbidity index (red), the competing risk model without the Charlson comorbidity index (blue) and QRISK3 (black) in women (left) and men (right). CR, competing risk model. CVD, cardiovascular disease. Charlson, Charlson comorbidity index. Observed risk is based on the Aalen-Johansen estimator, which accounts for competing mortality risk

**Fig. 2:**
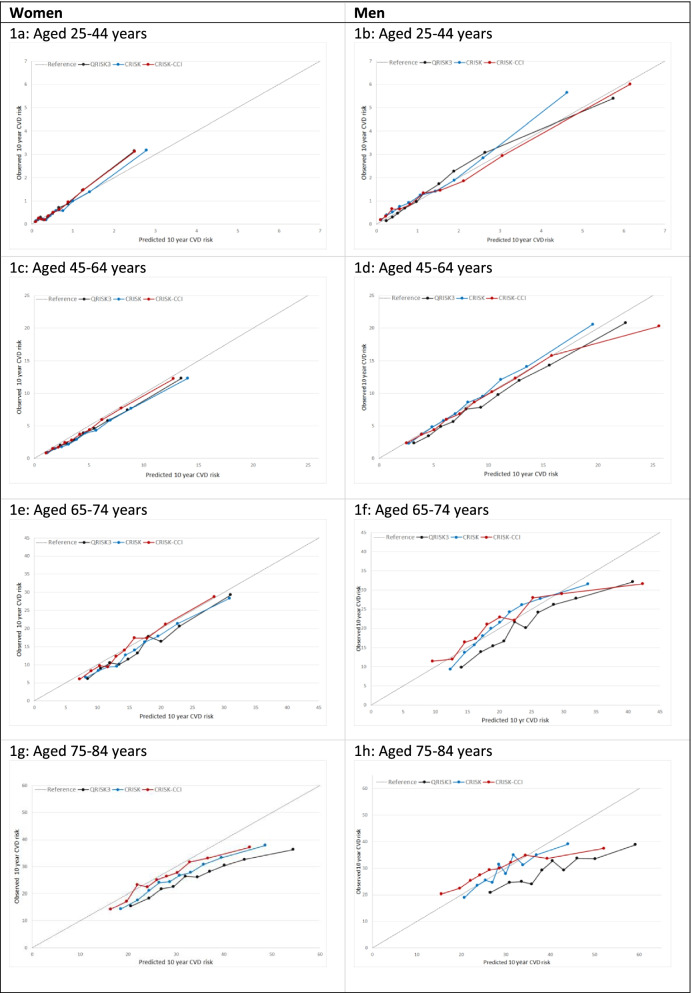
Calibration of CRISK-CCI (red), CRISK (blue) and QRISK3 (black) by age-group in women and men. CR, competing risk model. CVD, cardiovascular disease. Observed risk is based on the Aalen-Johansen estimator, which accounts for competing mortality risk. Ideal calibration lies on the reference line, below line is overprediction, and above line is underprediction

**Fig. 3:**
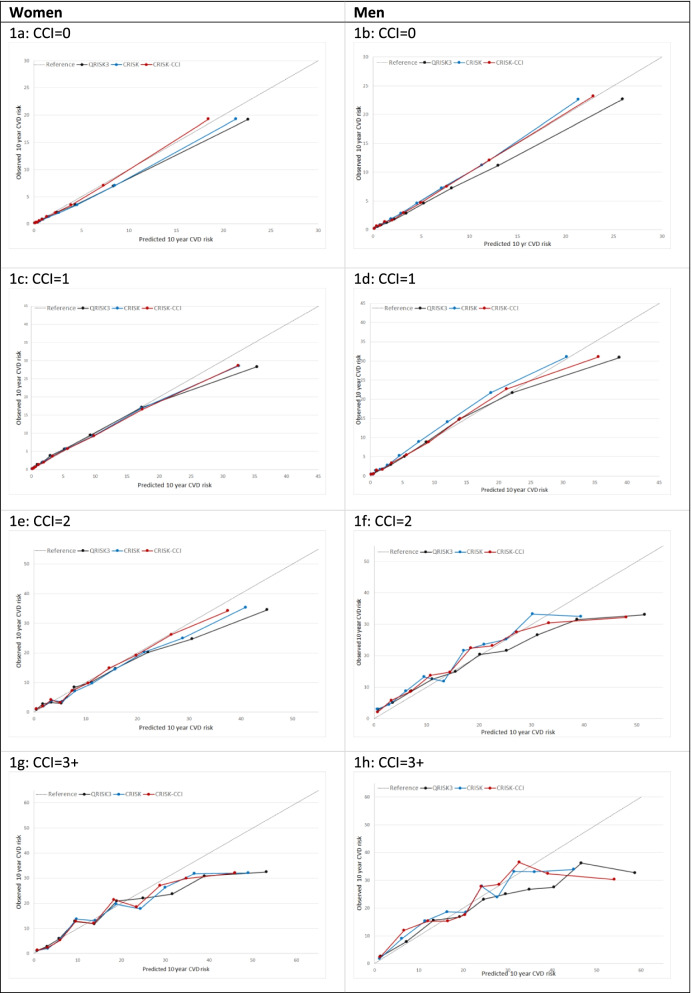
Calibration of CRISK-CCI (red), CRISK (blue) and QRISK3 (black) by CCI group in women and men. CR, competing risk model. CVD, cardiovascular disease. CCI, Modified Charlson comorbidity index. Observed risk is based on the Aalen-Johansen estimator, which accounts for competing mortality risk. Ideal calibration lies on the reference line, below line is overprediction, and above line is underprediction

In women overall, there was some overprediction with CRISK at higher levels of predicted risk but CRISK was better calibrated than QRISK3 overall, whilst calibration with CRISK-CCI was excellent (Fig. 1). In younger women, there was some underprediction with CRISK and CRISK-CCI that was similar to QRISK3 (Fig. 2). In older women, CRISK modestly over-predicted CVD risk, particularly at higher levels of predicted risk but was still better calibrated than QRISK3 whilst calibration with CRISK-CCI was excellent. In all CCI categories, there was some overprediction with each model at higher levels of predicted risk that was greatest with QRISK3 and least with CRISK-CCI (Fig. 3).

In men overall, calibration using CRISK-CCI was better than CRISK which showed some underprediction, whilst QRISK3 somewhat overpredicted CVD risk (Fig. 1). In younger men, there was some underprediction with CRISK and QRISK3, but calibration with CRISK-CCI was excellent (Fig. 2). In older men at lower levels of predicted risk, calibration with CRISK and CRISK-CCI was good, whilst there was overprediction with QRISK3. However, all models overpredicted risk at higher levels of predicted risk. In men with increasing CCI, there was some overprediction with each model at higher levels of predicted risk that was greatest QRISK3 and least with CRISK-CCI (Fig. 3).

### Reclassification of patients

The number and proportion of women and men reclassified by CRISK-CCI above and below each QRISK3 threshold is shown in Table 3. The proportion of patients reclassified by CRISK-CCI increased with higher categories of QRISK3 predicted risk. The proportion of people reclassified to a higher risk category by CRISK-CCI varied by risk category ranging from 0.7 to 9.7% in women and 2.8 to 25.2% in men. The proportion of people reclassified to a lower risk category by CRISK-CCI within each category of QRISK3 predicted risk ranged from 21.0 to 69.1% in women and 27.1 to 57.4% in men. At all levels of risk, CRISK-CCI reclassified more women and men to a lower rather than a higher predicted risk compared to QRISK3.

**Table 3:**
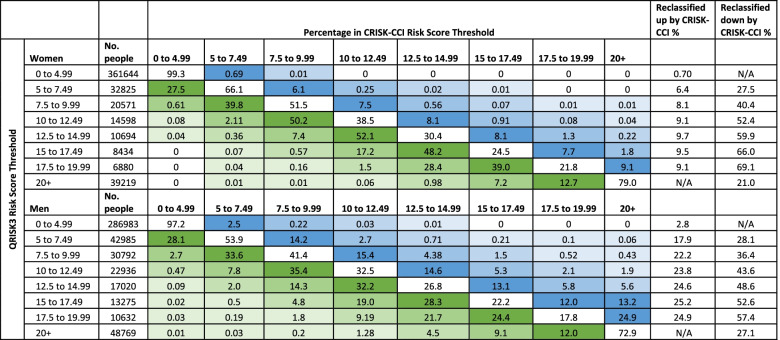
Percentage of men and women with particular categories of QRISK3 predicted risk in the validation cohort reclassified by CRISK with the Charlson comorbidity index

Numbers in the blue shaded cells are percentages of patients who are classified as at higher risk by CRISK-CCI than by QRISK3 at a particular risk threshold

Numbers in the green shaded cells are percentages of patients who are classified as lower by CRISK-CCI than by QRISK3 at a particular risk threshold. The darker the shaded colour the greater the row percentage reclassified as either higher risk (blue) or lower risk (green)

The number of patients recommended for treatment, number of events, and estimated NNT to prevent one new CVD event with CRISK-CCI and QRISK3 is shown in Table 4. In women at all risk thresholds, CRISK-CCI recommended fewer women for treatment, and the estimated NNT in those recommended for treatment was lower than for QRISK3 (20% threshold: NNT 23.9 for CRISK-CCI vs 25.8 for QRISK3, 10%: 34.2 vs 36.1, 7.5%: 39.5 vs 41.3). In men at all risk thresholds, CRISK-CCI recommended fewer men for treatment, and the estimated NNT was lower than for QRISK3 at the 10% (NNT 38.1 for CRISK-CCI vs 38.8 for QRISK3) and 7.5% (NNT 43.6 vs 44.2) thresholds, but higher at the 20% threshold (NNT 27.1 vs 26.5).

**Table 4:** Number of people recommended for treatment by QRISK3 and CRISK-CCI, observed event rate, and estimated number needed to treat to prevent an incident CVD event

Risk threshold	Number recommended for treatment (% of all people)	Number of CVD events (% of all events)	NNT to prevent 1 CVD event*
**Women**			
**20%**			
QRISK3	39219 (7.9%)	6084 (47.0%)	25.8
CRISK-CCI	31,811 (6.4%)	5344 (41.2%)	23.9
**10%**			
QRISK3	79,825 (16.1%)	8849 (68.3%)	36.1
CRISK-CCI	73,029 (14.8%)	8542 (65.9%)	34.2
**7.5%**			
QRISK3	100,396 (20.3%)	9718 (75.0%)	41.3
CRISK-CCI	93,855 (19.0%)	9498 (73.3%)	39.5
**Men**			
**20%**			
QRISK3	48,769 (10.3%)	7350 (45.1%)	26.5
CRISK-CCI	41,517 (8.8%)	6125 (37.6%)	27.1
**10%**			
QRISK3	112,632 (23.8%)	11,620 (71.3%)	38.8
CRISK-CCI	107,371 (22.7%)	11,272 (69.1%)	38.1
**7.5%**			
QRISK3	143,424 (30.3%)	12,967 (79.5%)	44.2
CRISK-CCI	138,352 (29.2%)	12,703 (77.9%)	43.6

*Assuming a 25% risk reduction with primary prevention statin treatment taken by all people recommended for treatment. *NNT* number needed to treat with statins. *CVD* cardiovascular disease

### Characteristics of reclassified patients

Compared to QRISK3 predictions, women and men reclassified above the 7.5%, 10%, and 20% thresholds by CRISK-CCI were younger and had lower SBP but higher mean BMI and a higher prevalence of current smoking compared to those reclassified below these thresholds by CRISK-CCI (Additional file 1: Table S6). Furthermore, men reclassified above the 7.5%, 10%, and 20% thresholds of QRISK3 by CRISK-CCI had higher mean total cholesterol to HDL ratio, whilst women reclassified in the same way had a higher prevalence of treated hypertension. For example, at the 10% threshold, women recommended for treatment by CRISK-CCI (but not recommended for treatment by QRISK3) had a mean age of 58.2 years vs 63.4 years in those recommended for treatment by QRISK3 vs not recommended for treatment by CRISK-CCI. In the same groups, total cholesterol to HDL ratio was 3.65 vs 3.77, mean BMI 30.3 kg/m^2^ vs 26.0 kg/m^2^, SBP 134.3 mmHg vs 140.0 mmHg, proportion of treated hypertension 37.0% vs 11.7%, and current smokers 39.7% vs 20.0%.

## Discussion

Both CRISK and CRISK-CCI had excellent discrimination at predicting incident CVD events, similar to QRISK3. In terms of calibration, CRISK modestly overpredicted at higher levels of predicted risk in but overprediction was less and calibration generally better than QRISK3. The inclusion of the Charlson Comorbidity Index score to predict non-cardiovascular mortality in CRISK-CCI further improved calibration. CRISK-CCI also resulted in a significant reclassification of patients into higher and lower thresholds of predicted risk that may inform primary prevention treatment recommendations. For example, among men who QRISK3 predicted to have a 7.5% to 9.99% predicted risk, 22.2% were reclassified above the 10% risk threshold, whilst in men who QRISK3 predicted to have a 10–12.49% risk, 43.6% were reclassified by CRISK-CCI below the 10% risk threshold. Overall, CRISK-CCI recommended fewer people for treatment and selected a population for treatment with a higher CVD risk and lower estimated NNT than QRISK3, with reclassification in women having the largest impact. Finally, at the 10% threshold, patients recommended for treatment by CRISK-CCI but *not* by QRISK3 were on average younger, had lower SBP, had higher BMI and a higher proportion of current smokers compared to patients recommended for treatment by QRISK3 but *not* by CRISK-CCI.

Strengths of the study includes adherence to methodological recommendations for risk prediction studies [20, 21], use of large representative population data, and use of a clean validation set to make comparisons with QRISK3. The study has several limitations. We incorporated the Charlson comorbidity score in CRISK to improve prediction of the competing risk of non-CVD death as it has been well validated. However, considering other predictors of mortality such as frailty might further improve prediction. As with other models using these type of UK clinical data, missing data for some variables was common. We used multiple imputation for these missing data, as has been done with QRISK3 and elsewhere, which relies on the assumption that all data are missing at random [10]. We had a higher proportion of missing data for total cholesterol to high density lipoprotein cholesterol ratio than in QRISK3 derivation to avoid including forward looking values in prediction. Whilst using a greater number of imputations would improve the relative efficiency to calculate a lower variance for the parameter estimates, the time taken to fit the Fine-Gray model on such a large data set meant this was not feasible. Despite this potential limitation, the relative efficiency using five imputations is considered good and significant differences were still observed. We also used a later index date (1 January 2004) for cohort entry than QRISK3 (which uses 1 January 1998), because we wished to better account for falling CVD incidence rates, rising statin prescribing trends over time, and improved data capture in primary care electronic health records. In this regard, deriving clinical prediction tools using increasingly historical data may result in bias [22]. Additionally, loss to follow-up due to deregistration was common, but we did not treat this as a competing risk and whether the assumption that patients censored because of deregistration have the same rate of events as those analysed is unknown. Finally, CRISK and CRISK-CCI were derived and validated in same dataset (i.e. internally validated), whereas QRISK3 is being externally validated as it was derived in a different dataset. External validation of both is required for a balanced comparison.

Two studies involving 4300 patients over the age of 65 years from the US Cardiovascular Health Study and one study involving all people over the age of 65 years from New Zealand have evaluated the impact of competing risk on CVD prediction [23–25]. These studies similarly noted only moderate discrimination of whole population CVD risk prediction tools in older adults, with a C-statistic of 0.63 for men and women from the US and 0.67 in a European replication cohort. One study involving all people ≥ 65 years from New Zealand similarly observed that their competing risk model was generally better calibrated compared to models derived using standard Cox regression methods [25]. Calibration in the US studies was dependent on cohort and sex with the direction of patient reclassification by the competing risk model among older people varying between studies. For example, in the study by Koller et al., the competing risk model reclassified more older people as higher risk in the US cohort, whilst in Europeans, a greater number were reclassified as lower risk [23]. Our study found that more patients were reclassified as lower risk compared to QRISK3 than were reclassified as higher risk compared to QRISK3. These studies differed from our study by including older adults only and did not use the same predictors to model CVD risk. In a large UK-based study evaluating a competing risk model against the now superseded QRISK2 tool, Van Staa et al. also observed larger differences between predicted and observed CVD risk among those with highest predicted risk, with QRISK2 overestimating the 10-year CVD risk by 2.2% in people aged > 65 years [7]. Our study also examined reclassification at different risk thresholds, finding that the impact of accounting for competing risks also varied with risk threshold. Differences in how patients are reclassified with CRISK-CCI likely relate to baseline differences in the rate of non-CVD death vs CVD events. A competing risk model should better account for such variability, which may be an important factor when considering the generalizability of such models across populations. Further research could examine this impact when models are either validated in other populations or when different models are applied to the same population. Indeed, two recently reported studies derived separate competing risk-adjusted CVD models for use in adults aged 40–69 years and 70 years and older in European populations that had similar discrimination in older adults. However, these models did not formally compare performance to that of existing prediction tools and only accounted for non-CVD mortality [26, 27].

For older people and people with high comorbidity, CVD prediction using QRISK3 has poor to fair discrimination and calibration. Prediction in these subgroups was better after accounting for competing mortality risk (CRISK) and better again when an additional validated predictor of total mortality (mCCI) was including in the model (CRISK-CCI). Competing risk models such as CRISK-CCI should therefore be considered for predicting CVD risk among older and multimorbid populations, if external validation in other datasets confirms better performance. The impact of CRISK and CRISK-CCI on CVD risk prediction varies by gender and by level of predicted risk and therefore depends upon the risk threshold chosen to inform clinical decision making. In some circumstances (such as the youngest age groups at low risk of both CVD and non-CVD death), accounting for competing risk will likely make little difference to recommendations. However, we believe that observed differences in model performance and patient reclassification in people close to treatment thresholds are large enough that CVD prediction models should either account for competing risk or robustly justify why they do not. Whatever prediction model is used, clinicians still need to use their judgement in making treatment recommendations based on a consideration of individual life expectancy and comorbidity [28]. Selecting people for primary prevention treatment is important despite falling costs of statin therapy because patient preference, treatment disutility, and the risk of side effects, however small, remain important factors. Even if models have similar overall discrimination and calibration, we demonstrate that the choice of model will make a difference to the type of patients recommended for treatment around key risk thresholds. CRISK and CRISK-CCI recommended fewer patients for treatment overall and those recommended for treatment had a lower estimated NNT to prevent a new CVD event consistent with better targeting of treatment and in particular recommended more younger people and fewer older people for treatment.

## Conclusions

We derived and validated a competing risk model to predict the 10-year risk of incident CVD events. CRISK and CRISK-CCI had similar discrimination to QRISK3 but were better calibrated, particularly among older people and those with non-CVD comorbidity. Overall, CRISK-CCI recommended fewer people for treatment, with a lower estimated NNT to prevent a CVD event. Clinicians should therefore consider using competing risk models for predicting new CVD events to guide primary prevention treatment decisions particularly in older people and those with non-CVD comorbidity.

## Supplementary Information

**Additional file 1: Figure S1.** Flow chart for cohort identification from CPRD GOLD. **Table S1.** Missing data handling for variables included in the model. **Table S3.** Incidence rates of cardiovascular disease per 1000 person years in derivation cohort **Table S4.** Adjusted subdistribution hazard ratios for CVD in women in the derivation cohort for CRISK-CCI. **Table S5.** Adjusted subdistribution hazard ratios for CVD in men in the derivation cohort for CRISK-CCI. **Table S6.** Characteristics of patients reclassified by CRISK-CCI in the validation cohort.

**Additional file 2: Table S2.** Follow-up status at 10 years by sex, age and co-morbidity in the derivation cohort.

## Data Availability

The data controller is the Clinical Practice Research Datalink (CPRD), and under the data licence granted, the authors are not allowed to share data. Researchers can apply to CPRD directly for access to the raw data.

## Abbreviations

BMI: Body mass index
CCI: Charlson comorbidity index
CPRD: Clinical Practice Research Datalink
CVD: Cardiovascular disease
HDL: High density lipoprotein
HES: Hospital Episode Statistics
ICD-10: International Classification of Disease 10th revision
ISAC: Independent Scientific Advisory Committee for MHRA database research
NNT: Number needed to treat
ONS: Office for National Statistics
SBP: Systolic blood pressure
sHRs: Subdistribution hazard ratios
TC: Total cholesterol
UK: United Kingdom
