# Supplementary material for: Predictive performance of a competing risk cardiovascular prediction tool CRISK compared to QRISK3 in older people and those with comorbidity: population cohort study
Source: BMC Med. 2022 May 4;20:152. doi: 10.1186/s12916-022-02349-6 (PMC9066924; doi:10.1186/s12916-022-02349-6)
Supplement: Supplementary file 1 — Additional file 1: Figure S1. Flow chart for cohort identification from CPRD GOLD. Table S1. Missing data handling for variables included in the model. Table S3. Incidence rates of cardiovascular disease per 1000 person years in derivation cohort Table S4. Adjusted subdistribution hazard ratios for CVD in women in the derivation cohort for CRISK-CCI. Table S5. Adjusted subdistribution hazard ratios for CVD in men in the derivation cohort for CRISK-CCI. Table S6. Characteristics of patients reclassified by CRISK-CCI in the validation cohort. [file 12916_2022_2349_MOESM1_ESM.docx]

**Predictive performance of a competing risk cardiovascular prediction tool CRISK compared to QRISK3 in older people and those with comorbidity: population cohort study**

**ADDITIONAL FILE 1**

Shona Livingstone, Division of Population Health and Genomics, University of Dundee, UK

Bruce Guthrie, Usher Institute, University of Edinburgh, UK

Peter T Donnan, Division of Population Health and Genomics, University of Dundee, UK

Alexander Thompson, Division of Population Health, Health Services Research & Primary Care, University of Manchester, UK

Daniel R Morales, ^1^Division of Population Health and Genomics, University of Dundee, UK. ^2^Department of Public Health, University of Southern Denmark, Denmark.

**Figure S1. Flow chart for cohort identification from CPRD GOLD.**

**Table S1: Missing data handling for variables included in the model.**

|  | How missingness was handled in analysis | Women  N=1484597  No (%) missing data | Men  N=1420176  No (%) missing data |
| --- | --- | --- | --- |
| Body mass index (BMI) | Imputed | 409464 (27.6) | 590513(41.6) |
| Total cholesterol:HDL cholesterol (TC:HDL) ratio | Imputed | 1260807(84.9) | 1207422(85.0) |
| Systolic blood pressure | Imputed | 252064 (17.0) | 489276(34.5) |
| Systolic blood pressure variability | Imputed | 695935 (46.9) | 1051621(74.0) |
| Smoking status | Imputed | 300216 (20.2) | 443494 (31.2) |
| Ethnicity | Assumed white | 309747(20.9) | 504698(35.5) |
| Socioeconomic status | Excluded | 2796 (0.2%) | 2671 (0.2%) |
| Complete data for BMI, TC:HDL ratio, systolic blood pressure, smoking status and ethnicity | NA | 170056 (11.5) | 153779 (10.8) |

**Table S3. Incidence rates of cardiovascular disease per 1000 person years in derivation cohort**

|  | **Women** |  |  | **Men** |  |  |
| --- | --- | --- | --- | --- | --- | --- |
| **Age, years** | **Incident CVD** | **Person years** | **Rate per 1000 person years (95% CI)** | **Incident CVD** | **Person years** | **Rate per 1000 person years (95% CI)** |
| 25-29 | 183 | 627737 | 0.29 (0.25, 0.34) | 192 | 601917 | 0.32 (0.28, 0.37) |
| 30-34 | 378 | 769509 | 0.49 (0.44, 0.54) | 640 | 762368 | 0.84 (0.78, 0.91) |
| 35-39 | 718 | 826019 | 0.87 (0.81, 0.94) | 1446 | 834211 | 1.73 (1.65, 1.82) |
| 40-44 | 1164 | 757893 | 1.54 (1.45, 1.63) | 2367 | 762609 | 3.10 (2.98, 3.23) |
| 45-49 | 1481 | 625953 | 2.37 (2.25, 2.49) | 3206 | 611767 | 5.24 (5.06, 5.42) |
| 50-54 | 1835 | 541724 | 3.39 (3.24, 3.55) | 3714 | 498186 | 7.46 (7.22, 7.70) |
| 55-59 | 2511 | 504069 | 4.98 (4.79, 5.18) | 4614 | 436354 | 10.57 (10.27,10.88) |
| 60-64 | 2705 | 339041 | 7.98 (7.68, 8.28) | 4163 | 273034 | 15.25 (14.79,15.71) |
| 65-69 | 3241 | 258133 | 12.56 (12.13,12.99) | 4093 | 193741 | 21.13 (20.50,21.78) |
| 70-74 | 4100 | 202816 | 20.22 (19.61,20.84) | 4114 | 137928 | 29.83 (28.94,30.74) |
| 75-80 | 4751 | 158221 | 30.03 (29.20,30.88) | 3857 | 95114 | 40.55 (39.32,41.82) |
| 80-84 | 5234 | 117845 | 44.41 (43.25,45.61) | 2971 | 56672 | 52.42 (50.62,54.29) |
| Total | 28301 | 5728960 | 4.94 (4.88, 5.00) | 35377 | 5263900 | 6.72 (6.65, 6.79) |

CVD=Cardiovascular disease. 95%CI=95% confidence interval.

**Table S4. Adjusted subdistribution hazard ratios for CVD in women in the derivation cohort for CRISK-CCI.**

| **Predictor** | **Subdistribution Hazard Ratio (95% CI)** |
| --- | --- |
| (Age in years/10)^0.5^ - 2.1163 | 65.8 (51.5,83.9) |
| (BMI/10)^2^ - 7.0332 | 1.03 (1.02,1.05) |
| (SBP/10)- 12.5032 | 1.11 (1.06,1.16) |
| log(TC:HDL) - 1.2069 | 1.47 (1.28,1.67) |
| (Townsend score + 3.8101)^0.5^ -1.620811 | 1.31 (1.19,1.44) |
| Variance in SBP - 9.5910 | 1.01 (1.01,1.02) |
| Atrial fibrillation | 4.98 (2.52,9.83) |
| Atypical antipsychotics | 1.09 (0.71,1.68) |
| Corticosteroid use | 2.16 (1.40,3.26) |
| Migraine | 1.50 (1.21,1.86) |
| Rheumatoid arthritis | 1.31 (1.01,1.69) |
| Chronic kidney disease (stage 3, 4 or 5) | 1.79 (0.59,5.43) |
| Serious mental illness | 1.22 (1.06,1.40) |
| Systemic lupus erythematosus (SLE) | 1.74 (0.60,4.98) |
| Treated hypertension | 1.48 (1.13,1.94) |
| Type 1 diabetes | 2.69 (1.29,5.58) |
| Type 2 diabetes | 2.19 (1.30,3.71) |
| Family history of CHD in 1^st^ degree relative <60 years | 1.27 (1.02,1.59) |
| Smoking status |  |
| Non smoker | 1 |
| Former smoker | 1.32 (1.07,1.63) |
| Light smoker | 2.05 (1.65,2.54) |
| Moderate smoker | 2.25 (1.82,2.79) |
| Heavy smoker | 2.62 (2.10,3.28) |
| Ethnicity | 1 |
| Indian, Pakistani or Bangladeshi | 1.53 (1.10,2.12) |
| OtherAsian | 1.14 (0.47,2.76) |
| Black-Caribbean or Black African | 0.97 (0.61,1.52) |
| Other | 1.07 (0.77,1.49) |
| Charlson comorbidity index |  |
| 0 | 1 |
| 1 | 1.22 (1.11,1.34) |
| 2 | 1.11 (0.96,1.29) |
| ≥3 | 1.18 (0.94,1.49) |
| Interactions with age term |  |
| Age term – Atrial fibrillation | 0.22 (0.08,0.63) |
| Age term - Corticosteroid use | 0.38 (0.17,0.81) |
| Age term - Migraine | 0.60 (0.35,1.01) |
| Age term - CKD | 0.46 (0.07,3.18) |
| Age term – Treated hypertension | 0.73 (0.46,1.15) |
| Age term – Type 1 diabetes | 0.61 (0.14,2.63) |
| Age term – Type 2 diabetes | 0.43 (0.17,1.07) |
| Age term – Family history of CHD | 0.81 (0.45,1.44) |
| Age term - Former smoker | 0.84 (0.57,1.24) |
| Age term - Light smoker | 0.67 (0.44,1.03) |
| Age term - Moderate smoker | 0.55 (0.35,0.87) |
| Age term – Heavy smoker | 0.44 (0.26,0.73) |
| Age term – BMI term | 0.97 (0.94,1.00) |
| Age term – SBP term | 0.92 (0.85,0.99) |
| Age term – Townsend term | 0.71 (0.59,0.85) |
| Age term - SLE | 0.88 (0.06,12.2) |

CI, confidence interval. BMI, body mass index (Kg/m2). SBP, systolic blood pressure in mmHg. TC: HDL, Total cholesterol:HDL-c ratio. SLE, Systemic lupus erythematosus. CHD, coronary heart disease. CKD, Chronic kidney disease (stage 3, 4 or 5). Baseline 10 year CIF of 0.712743% corresponding to the following baseline characteristics: age 44-45 years, BMI 26.5 kg/m^2^, SBP 125 mmHg, TC:HDL 3.3 mmol/L, Townsend score of -1.183, variance in BP 9.6, white or assumed white ethnicity, non-smoker and none of the above conditions. The sHRs describe the direction but not the magnitude of the effect of predictors on the CIF and are included for model reproducibility.

**Table S5. Adjusted subdistribution hazard ratios for CVD in men in the derivation cohort for CRISK-CCI.**

| **Predictor** | **Subdistribution Hazard Ratio (95% CI)** |
| --- | --- |
| (Age in years/10)^-0.5^ -0.4888 | 7.66x10^-10^ (2.05x10^-10^, 2.87x10^-9^) |
| (BMI/10) - 2.6514 | 1.29 (1.14,1.46) |
| (SBP/10) -13.0309 | 1.12 (1.07,1.16) |
| log(TC:HDL) -1.4124 | 1.54 (1.37,1.74) |
| (Townsend score+3.8101)^0.5^ - 1.6479 | 1.20 (1.10,1.30) |
| (Variance in SBP+0.0001)^-0.5^-1.6701 | 1.00 (1.00,1.00) |
| Atrial fibrillation | 3.92 (1.71,8.98) |
| Atypical antipsychotics | 1.12 (0.78,1.60) |
| Corticosteroid use | 1.82 (1.12,2.98) |
| Impotence | 1.25 (0.87,1.80) |
| Migraine | 1.48 (1.12,1.97) |
| Rheumatoid arthritis | 1.19 (0.85,1.68) |
| Chronic kidney disease (stage 3, 4 or 5) | 2.13 (1.19,3.80) |
| Serious mental illness | 1.22 (1.04,1.44) |
| Systemic lupus erythematosus (SLE) | 0.381 (0.05,3.06) |
| Treated hypertension | 1.70 (1.28,2.25) |
| Type 1 diabetes | 2.17 (0.71,6.70) |
| Type 2 diabetes | 1.87 (1.11,3.15) |
| Family history of CHD in 1^st^ degree relative <60 years | 1.64 (1.32,2.02) |
| Smoking status |  |
| Non smoker | 1 |
| Former smoker | 1.11 (0.93,1.33) |
| Light smoker | 1.84 (1.48,2.29) |
| Moderate smoker | 2.01 (1.68,2.42) |
| Heavy smoker | 2.59 (2.19,3.06) |
| Ethnicity | 1 |
| Indian, Pakistani or Bangladeshi | 1.75 (1.30,2.36) |
| OtherAsian | 1.58 (0.92,2.71) |
| Black-Caribbean or Black African | 0.71 (0.46,1.10) |
| Other | 1.17 (0.89,1.53) |
| Charlson comorbidity index |  |
| 0 | 1 |
| 1 | 1.17 (1.07,1.28) |
| 2 | 1.19 (1.02,1.38) |
| ≥3 | 1.17 (0.93,1.46) |
| Interactions with age term |  |
| Age term – Atrial fibrillation | 2.92 (2.17, 3.93x10^6^) |
| Age term – Impotence | 20.1 (0.18,2.21x10^3^) |
| Age term - Corticosteroid use | 3.71 (0.07,192) |
| Age term - Migraine | 9.65 (0.28,336) |
| Age term - CKD | 259 (1.40,4.81x10^4^) |
| Age term – Treated hypertension | 21.4 (1.45,314) |
| Age term – Type 1 diabetes | 2.77 (2.23x10^-7^, 3.42x10^7^) |
| Age term – Type 2 diabetes | 31.5 (0.24,4.14x10^3^) |
| Age term – Family history of CHD | 13.7 (0.73,256) |
| Age term - Former smoker | 1.73 (0.265,11.3) |
| Age term - Light smoker | 17.6 (1.5,207) |
| Age term - Moderate smoker | 44.2 (5.19,376) |
| Age term – Heavy smoker | 120 (14.9,961) |
| Age term – BMI term | 3.51 (0.891,13.8) |
| Age term – SBP term | 1.48 (0.953,2.29) |
| Age term – Townsend term | 2.71 (1.1,6.67) |

CI, confidence interval. BMI, body mass index (Kg/m2). SBP, systolic blood pressure in mmHg. TC: HDL, Total cholesterol:HDL-c ratio. SLE, Systemic lupus erythematosus. CHD, coronary heart disease. CKD, Chronic kidney disease (stage 3, 4 or 5). Baseline 10 year CIF of 1.3133% corresponding to the following baseline characteristics: age 41-42 years, BMI 26.5 kg/m^2^, SBP 130 mmHg, TC:HDL 4.1 mmol/L, Townsend score of -1.095, variance in SBP 0.4, white or assumed white ethnicity, non-smoker and none of the above conditions. The sHRs describe the direction but not the magnitude of the effect of predictors on the CIF and are included for model reproducibility.

**Table S6. Characteristics of patients reclassified by CRISK-CCI in the validation cohort**

| **Groups where CRISK-CCI and QRISK3 predicted risk differed*** | **Mean (SD) age**  **(years)** | **Mean (SD) TC:HDL ratio** | **Mean (SD) Systolic Blood Pressure (mmHg)** | **Treated**  **hypertension** **(%)** | **Current**  **smokers (%)** | **Mean (SD) BMI**  **(kg/m^2^)** |
| --- | --- | --- | --- | --- | --- | --- |
| **Women** |  |  |  |  |  |  |
| 20% threshold |  |  |  |  |  |  |
| QRISK3 below, CRISK-CCI above | 66.3 (65.9,66.8) | 3.69 (3.63,3.76) | 140.1 (139.0,141.2) | 66.3 (63.1,69.6) | 36.8 (33.4,40.2) | 30.7 (30.2, 31.3) |
| QRISK3 above, CRISK-CCI below | 73.5 (73.4,73.6) | 3.72 (3.69,3.74) | 145.3 (145.0,145.7) | 18.0 (17.1,18.8) | 15.0 (14.1,15.8) | 25.1 (24.9, 25.2) |
| 10% threshold |  |  |  |  |  |  |
| QRISK3 below, CRISK-CCI above | 58.2 (57.9,58.5) | 3.65 (3.61,3.70) | 134.3 (133.5,135.0) | 37.0 (34.8,39.3) | 39.7 (37.3,42.1) | 30.3 (29.9, 30.7) |
| QRISK3 above, CRISK-CCI below | 63.4 (63.2,63.5) | 3.77 (3.75,3.80) | 140.0 (139.7,140.4) | 11.7 (11.1,12.4) | 20.0 (19.0,21.1) | 26.0 (25.9, 26.1) |
| 7.5% threshold |  |  |  |  |  |  |
| QRISK3 below, CRISK-CCI above | 55.0 (54.8,55.3) | 3.62 (3.58,3.66) | 131.8 (131.1,132.4) | 25.0 (23.2,26.8) | 41.9 (39.8,44.0) | 29.7 (29.4, 30.1) |
| QRISK3 above, CRISK-CCI below | 59.8 (59.7,60.0) | 3.71 (3.69,3.74) | 137.8 (137.5,138.1) | 10.5 (9.8,11.1) | 19.5 (18.5,20.5) | 26.1 (25.9, 26.2) |
| **Men** |  |  |  |  |  |  |
| 20% threshold |  |  |  |  |  |  |
| QRISK3 below, CRISK-CCI above | 55.9 (55.7,56.1) | 5.72 (5.69,5.76) | 136.9 (136.5,137.3) | 22.6 (21.6,23.7) | 40.4 (39.1,41.7) | 30.2 (30.1, 30.4) |
| QRISK3 above, CRISK-CCI below | 70.1 (70.0,70.2) | 3.47 (3.45,3.49) | 142.0 (141.7,142.3) | 17.2 (16.5,17.8) | 23.1 (22.0,24.2) | 25.0 (24.9, 25.1) |
| 10% threshold |  |  |  |  |  |  |
| QRISK3 below, CRISK-CCI above | 49.1 (48.9,49.2) | 5.44 (5.41,5.47) | 131.3 (131.0,131.6) | 7.6 (7.0, 8.1) | 41.5 (40.4,42.6) | 29.5 (29.4, 29.6) |
| QRISK3 above, CRISK-CCI below | 58.6 (58.5,58.7) | 3.48 (3.46,3.49) | 137.0 (136.8,137.3) | 9.5 (9.0,10.0) | 28.2 (27.1,29.2) | 25.2 (25.1, 25.2) |
| 7.5% threshold |  |  |  |  |  |  |
| QRISK3 below, CRISK-CCI above | 46.2 (46.1,46.3) | 5.36 (5.33,5.39) | 129.4 (129.2,129.7) | 3.6 (3.2, 4.0) | 43.3 (42.1,44.5) | 29.2 (29.1, 29.3) |
| QRISK3 above, CRISK-CCI below | 54.8 (54.7,54.9) | 3.48 (3.46,3.50) | 134.9 (134.6,135.2) | 7.6 (7.2, 8.0) | 28.0 (27.0,29.1) | 25.2 (25.1, 25.3) |

* Groups where scores agree not shown. TC:HDL=total cholesterol:high density cholesterol ratio. Body mass index. BP= blood pressure.
