# Supplementary material for: Predictive performance of a competing risk cardiovascular prediction tool CRISK compared to QRISK3 in older people and those with comorbidity: population cohort study
Source: BMC Med. 2022 May 4;20:152. doi: 10.1186/s12916-022-02349-6 (PMC9066924; doi:10.1186/s12916-022-02349-6)
Supplement: Supplementary file 2 — Additional file 2: Table S2. Follow-up status at 10 years by sex, age and co-morbidity in the derivation cohort. [file 12916_2022_2349_MOESM2_ESM.docx]

**Table S2. Follow-up status at 10 years by sex, age and co-morbidity in the derivation cohort.**

|  | **No. entering study cohort** | **No. with at least 10 yrs follow-up** | **No. with non-fatal CVD** | **No with CVD death** | **No. censored after non-CVD death** | **No. censored after starting statin** | **No. censored after deregistration or study end (31/3/16)** |
| --- | --- | --- | --- | --- | --- | --- | --- |
| All women | 989732 | 232819 (23.5) | 22710 (2.3) | 3379 (0.3) | 27221 (2.8) | 85517 (8.6) | 618086 (62.4) |
| All men | 946784 | 194625 20.6) | 28480 (3.0) | 4351 (0.5) | 25446 (2.7) | 97236 (10.3) | 596646 (63.0) |
| Women |  |  |  |  |  |  |  |
| Aged 25-44 | 542145 | 120141 (22.2) | 2042 (0.4) | 79 (0.0) | 2165 (0.4) | 9397 (1.7) | 408321 (75.3) |
| Aged 45-64 | 310371 | 87807 (28.3) | 7236 (2.3) | 463 (0.1) | 7400 (2.4) | 45837 (14.8) | 161628 (52.1) |
| Aged 65-74 | 80831 | 17405 (21.5) | 5946 (7.4) | 790 (1.0) | 6301 (7.8) | 21451 (26.5) | 28938 (35.8) |
| Aged 75-84 | 56385 | 7466 (13.2) | 7486 (13.3) | 2047 (3.6) | 11355 (20.1) | 8832 (15.7) | 19199 (34.0) |
| CCI score |  |  |  |  |  |  |  |
| 0 | 791395 | 189520 (23.9) | 14601 (1.8) | 1966 (0.2) | 14885 (1.9) | 57871 (7.3) | 512552 (64.8) |
| 1 | 153590 | 35860 (23.3) | 5308 (3.5) | 885 (0.6) | 6152 (4.0) | 19052 (12.4) | 86333 (56.2) |
| 2 | 34250 | 6135 (17.9) | 1966 (5.7) | 360 (1.1) | 4114 (12.0) | 6531 (19.1) | 15144 (44.2) |
| 3+ | 10497 | 1304 (12.4) | 835 (8.0) | 168 (1.6) | 2070 (19.7) | 2063 (19.7) | 4057 (38.6) |
| Men |  |  |  |  |  |  |  |
| Aged 25-44 | 544082 | 110581 (20.3) | 3773 (0.7) | 316 (0.1) | 2831 (0.5) | 16775 (3.1) | 409806 (75.3) |
| Aged 45-64 | 305506 | 69294 (22.7) | 13083 (4.3) | 1411 (0.5) | 8090 (2.6) | 57763 (18.9) | 155865 (51.0) |
| Aged 65-74 | 64301 | 11096 (17.3) | 6577 (10.2) | 1108 (1.7) | 6351 (9.9) | 17913 (27.9) | 21256 (33.1) |
| Aged 75-84 | 32895 | 3654 (11.1) | 5047 (15.3) | 1516 (4.6) | 8174 (24.8) | 4785 (14.5) | 9719 (29.5) |
| CCI score |  |  |  |  |  |  |  |
| 0 | 781665 | 164303 (21.0) | 20388 (2.6) | 2852 (0.4) | 15339 (2.0) | 70131 (9.0) | 508652 (65.1) |
| 1 | 134497 | 26674 (19.8) | 5461 (4.1) | 927 (0.7) | 5207 (3.9) | 20047 (14.9) | 76181 (56.6) |
| 2 | 23133 | 2994 (12.9) | 1891 (8.2) | 375 (1.6) | 3141 (13.6) | 5396 (23.3) | 9336 (40.4) |
| 3+ | 7489 | 654 (8.7) | 740 (9.9) | 197 (2.6) | 1759 (23.5) | 1662 (22.2) | 2477 (33.1) |
